# The induction of CCN2 by TGFβ1 involves Ets-1

**DOI:** 10.1186/ar1890

**Published:** 2006-01-16

**Authors:** Jonathan P Van Beek, Laura Kennedy, Jason S Rockel, Suzanne M Bernier, Andrew Leask

**Affiliations:** 1 CIHR Group in Skeletal Development and Remodeling, Schulich School of Medicine and Dentistry, Dental Sciences Building, The University of Western Ontario, London, ON N6A 5C1, Canada

## Abstract

CCN2 is encoded by an immediate-early gene induced in mesenchymal cells during the formation of blood vessels, bone and connective tissue. It plays key roles in cell adhesion and migration, as well as matrix remodeling. CCN2 is overexpressed in fibrosis, arthritis and cancer; thus, an understanding of how to control CCN2 expression is likely to have importance in developing therapies to combat these pathologies. Previously, we found that the promoter sequence GAGGAATG is important for *Ccn2 *gene regulation in NIH 3T3 fibroblasts. In this report, we show that this sequence mediates activation of the CCN2 promoter by the ETS family of transcription factors. Endogenous Ets-1 binds this element of the CCN2 promoter, and dominant negative Ets-1 and specific Ets-1 small interfering RNA block induction of CCN2 expression by TGFβ. In the absence of added TGFβ1, Ets-1, but not the related fli-1, synergizes with Smad 3 to activate the CCN2 promoter. Whereas the ability of transfected Ets-1 to activate the CCN2 promoter is dependent on protein kinase C (PKC), Ets-1 in the presence of co-transfected Smad3 does not require PKC, suggesting that the presence of Smad3 bypasses the requirement of Ets-1 for PKC to activate target promoter activity. Our results are consistent with the notion that Smad3 and Ets-1 cooperate in the induction of the CCN2 promoter by TGFβ1. Antagonizing Ets-1 might be of benefit in attenuating CCN2 expression in fibrosis, arthritis and cancer, and may be useful in modulating the outcome of these disorders.

## Introduction

CCN2 (connective tissue growth factor) is a member of the CCN family of matricellular proteins that share a similar predicted structure [1]. It is thought to comprise four protein modules sharing identity with insulin-like growth factor binding proteins, Von Willebrand factor, thrombospondin, and a cysteine knot-containing family of growth regulators [2]. CCN2 is a secreted protein [3] and as such promotes cell migration, angiogenesis and fibrotic responses *in vivo *and *in vitro *[2] through a unique integrin- and heparin sulfate proteoglycan-dependent mechanism [4,5]. CCN2 is expressed in mesenchymal cells during development, and mice possessing a deleted *Ccn2 *gene die soon after birth due to an inability to breathe caused by a failure in rib cage ossification, angiogenesis and matrix remodeling [6]. Embryonic fibroblasts cultured from CCN2-deficient animals show reduced signaling responses to adhesion and impaired stress fiber formation on fibronectin, suggesting that a physiological role of CCN2 is to potentiate interaction of cells with matrix [5]. Indeed, a principal, if not primary, role of CCN2 is to modulate adhesive signaling [3-5]. Consistent with a role for CCN2 in tissue formation and remodeling, CCN2 is induced during angiogenesis, wound healing and tissue repair [6], and is constitutively overexpressed in cancer, atherosclerosis, arthritis and fibrosis [2,6]. Gaining insight into how CCN2 expression is controlled is likely to improve the understanding of the molecular basis of these pathological conditions, as well as to identify potential new avenues for therapeutic interventions for these disorders.

The cell type in which CCN2 expression has been most extensively studied is the fibroblast. The potent pro-fibrotic protein transforming growth factor (TGF)β induces CCN2 expression in dermal fibroblasts, but not in dermal keratinocytes [7-9]. TGFβ induction of CCN2 mRNA in fibroblasts occurs in an immediate-early fashion, within 30 minutes of TGFβ treatment [7,8]. This induction requires Smad3, protein kinase C (PKC) and ras/MEK/ERK [9-11]. In fibroblasts, the TGFβ-mediated induction of CCN2 is antagonized by AP-1/JNK, suggesting that a balance between MEK/ERK and JNK activation is important in controlling CCN2 expression [9]. The induction of the CCN2 promoter also requires a tandem repeat of the nucleotides GAGGAATGG, which binds factors enriched in fibroblasts relative to keratinocytes, suggesting that this element controls the cell type-restricted response of the CCN2 promoter to TGFβ [9]. This element has previously been identified and mapped using extensive point mutational analysis [9]. However, the identities of the factors binding this element have not been elucidated, nor has the potential for control of CCN2 expression by different transcription factors interacting with this element been clarified.

Ets proteins, which bind the promoter element GGAA/T, are a large family of transcription factors of which several members are expressed in a tissue- and cell type-restricted fashion [12,13]. Because of this diversity, multiple Ets factors may be able to control the same target genes, albeit to different outcomes. In addition, functional antagonism between different Ets factors and between Ets and other transcription factors has been observed and the combination of Ets proteins and their coactivators expressed in a particular cell type is likely to contribute to the cell-type expression of target genes in normal and pathological states, resulting in distinct pathological consequences. Ets family members regulate the expression of several genes encoding extracellular matrix and adhesive proteins as well as enzymes involved in matrix degradation [12,13]. Upon tissue injury, Ets-1 activity is transiently induced in endothelial cells, smooth muscle cells and fibroblasts during the early stages of tissue remodeling (for example, in the early phase of ulcer healing) or immediately after mechanical injury of the vessel wall [14]. Although Ets-1 DNA binding activity is increased in scleroderma fibroblasts [15], the Ets family member Fli-1 has reduced expression in this cell type [16]; however, the consequences of altering the Ets-1/Fli-1 ratios on mesenchymal biology has yet to be fully appreciated. Ets-1 is overexpressed in synovial fibroblasts from arthritis patients [17] and is induced during physiological and pathological angiogenesis [13]. The precise target genes, and physiological effect, of Ets family members in remodeling and repair of connective tissue and associated pathologies is still under much scrutiny.

In this study, we evaluate the hypothesis that the expression of CCN2 can be regulated through the activity of Ets-1. Our results reveal new insights into the control of CCN2 expression in fibroblasts, and the role of Ets-1 in fibroblast biology. Our results have implications for the function of CCN2 in physiological tissue repair and in pathologies of the extracellular matrix.

## Materials and methods

### Cell culture, transfections and DNA constructs

NIH 3T3 fibroblasts were purchased (ATCC Manassas, VA, USA) and cultured in DMEM, 10% calf serum penicillin/streptomycin (Invitrogen, Carlsbad, CA, USA) as described by the supplier. Cells were transfected using polyfect (QiagenValencia, CA, USA) as described by the manufacturer and as previously described [9,10,18,19]. Briefly, NIH 3T3 cells (3 × 10^5 ^cells/well) were placed into 6-well plates. The next day, cells were transfected with CCN2 promoter/secreted enhanced alkaline phosphatase (SEAP) reporter expression vectors (0.5 μg DNA/well) as previously described [9,10,18,19]. Promoter/reporter constructs contained CCN2 promoter fragments spanning nucleotides -805 to +17 (-805), -244 to +17 (-244) and -86 to +17 (-86). In addition, CCN2 promoter constructs used contained mutations in the Smad element (TCAGA to GGATC) and GGAA (GGAAT to TCCCG) element introduced into the CCN2 promoter between nucleotides -805 to +17, but were otherwise identical to construct -805. CCN2 promoter constructs were co-transfected with expression vectors (1 μg DNA/well) encoding Ets-1 and Fli-1 (Philip Marsden, University of Toronto), dominant negative Ets-1 (Hiroshi Sato, Kanazawa University) or Smad3 (Joan Massague, Sloan-Kettering) when appropriate. Cells were also co-transfected with a control CMV-β-galactosidase vector (0.25 μg/well; Clontech, Palo Alto, CA, USA as an internal transfection control. Transfection was performed in serum-free DMEM, and all cells were cultured for an additional 24 hours in DMEM, 0.5% calf serum, followed by a further incubation for 24 hours in the presence or absence of 4 ng/ml TGFβ1 (R and D Systems, Minneapolis, MN, USA) or bisindolymaleimide I (10 μM, Calbiochem, La Jolla, CA USA). Promoter assays were then performed (Applied Biosystems, Foster City, CA USA). Reporter (SEAP) expression was adjusted for differences in β-galactosidase expression and expressed as average ± standard deviation of at least three replicates and at least two independent trials. Representative experiments are shown. Statistical analysis (*p *< 0.05) was performed using the Student's *t *test.

### Gel shift analysis

Nuclear extracts were prepared using a kit (Pierce, Rockford, IL, USA) and protein concentration was determined (Bio-Rad, Hercules, CA, USA). Gel shifts were performed using 5 μg of nuclear extract as described [9]. A double-stranded annealed oligomer spanning nucleotides -126 to -77 of the Ccn2 promoter (Sigma-Genosys, St Lois, MO USA) waslabeled with ^32^P-ATP (New England Nuclear, Montreal, QC, Canada) using polynucleotide kinase (New England Biolabs Beverley MA USA). As DNA competitors, 100-fold molar excess of either unlabeled wild-type probe or oligomers containing either a consensus Ets or NFκB binding element (Santa Cruz Biotechnology, Santa Cruz, CA, USA) were used. For antibody competition assays, 1 μl of anti-Ets-1, anti-Fli-1, anti-Sp1 or anti-Elk-1 antibody (Santa Cruz Biotechnology) was added to the binding mixture for 1 hour prior to addition of probe. As previously described [21], all components of the DNA binding reaction were combined and incubated at room temperature, prior to addition of radiolabeled probe (60,000 cpm/reaction). After 30 minutes of incubation at room temperature, the binding reaction was subjected to non-denaturing polyacrylamide gel electrophoresis in 0.5× TBE, 20 mA. Gels were dried, and subjected to autoradiography to detect protein/DNA complexes, which were quantified using densitometry (Alpha Innotech, San Leandro, CA, USA).

### siRNA, Western blot and immunofluorescence analysis

Human dermal fibroblasts (ATCC) were transfected with either 50 nM control small interfering RNA (siRNA; cyclophilin, Dharmacon, Lafayette, CO, USA) or Ets-1 or Fli-1 siRNA (SMART Pool, Dharmacon) using Dharmafect 1, as described by the manufacturer. After a 24 hour incubation in serum-free DMEM, cells were incubated in the presence or absence of 4 ng/ml TGFβ1 for an additional 24 hours. Cell extracts were subjected to western blot analyses with anti-CCN2, anti-Ets-1, anti-Fli-1 and anti-β-actin (Sigma, St Louis, MO, USA) antibodies. Cells were also fixed in 4% paraformaldehyde, 15 minutes, room temperature, and indirect immunofluorescence analysis to detect CCN2 was performed using an anti-CCN2 antibody (Santa Cruz Biotechnology) and a Texas Red-conjugated secondary antibody (Jackson Immunoresearch, West Grove, PA, USA) as previously described [3]. Cells were counterstained with DAPI (1 μg/ml, 10 minutes; Molecular Probes, Eugene, OR, USA) and images were captured using a Leica microscope and Q Imaging software (Burnaby, BC, Canada).

## Results

### ETS family members activate the CCN2 promoter through GAGGAATG

**Figure 1:**
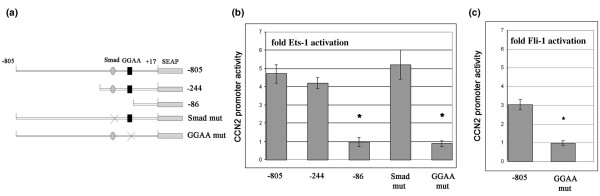
ETS family members activate the CCN2 promoter. **(a) **Schematic diagram of CCN2 promoter constructs used for this study: -805, construct containing -805 to +17 of the CCN promoter; -244, construct containing -244 to +17; -86, construct containing -86 to +17; Smadmut, construct containing mutated Smad element in the context of -805 to +17; GGAAmut, construct containing mutated GGAA element in the context of -805 to +17 [9,10,18,19]. Characterization of the CCN2 promoter response to **(b) **Ets-1 or **(c) **Fli-1. Different CCN2 promoter/reporter constructs, as indicated, were transfected into fibroblasts with either empty expression vector or expression vector encoding Ets-1, as described in Materials and methods. Fold increase with overexpression of **(b) **Ets-1 or **(c) **Fli-1, relative to the activity observed in the presence of empty control expression vector is shown. Average ± standard deviation (N = 6) of a representative experiment is shown (*p* < 0.05; asterisks indicate significantly modified by overexpression of transcription factor). Reporter activity was adjusted for differences in transfection efficiencies among samples using a control β-galactosidase expression vector.

To assess if the CCN2 promoter was responsive to Ets-1, we transfected NIH 3T3 fibroblasts with a full-length CCN2 promoter/SEAP reporter construct (driven by nucleotides -805 to +17 of the CCN2 promoter; Figure 1a) in the presence of either expression vector encoding Ets-1 or empty expression vector. We found that overexpression of Ets-1 increased activity of the full-length CCN2 promoter (Figure 1). To map the Ets-1 response element in the CCN2 promoter, we transfected NIH 3T3 fibroblasts with CCN2 promoter/reporter constructs that contained different segments of the CCN2 promoter (Figure 1a). We found that, whereas a SEAP reporter gene driven by nucleotides -244 to +17 responded to Ets-1, a construct containing nucleotides -86 to +17 no longer responded to Ets-1 (Figure 1b, compare -805, -244 and -86). To further delineate the elements of the CCN2 promoter required for the CCN2 promoter to respond to Ets-1, we transfected into NIH 3T3 cells CCN2 promoter constructs containing point mutations within regions of the CCN2 promoter previously shown to be important for its regulation. We found that mutation of the Smad element [19] of the CCN2 promoter did not significantly affect the ability of Ets-1 to activate it (Figure 1b). Conversely, mutation of the consensus Ets binding motif GGAA within the transcription enhancing factor (TEF) binding element GAGGAATG located between -91 to -84, previously shown to be important for basal and TGFβ-induced CCN2 expression [9], abolished the ability of the CCN2 promoter to respond to Ets-1 (Figure 1b). To extend these results, we found that the ETS family member Fli-1 could activate the CCN2 promoter in fibroblasts in a fashion dependent on the GGAA motif (Figure 1c). With respect to basal CCN2 promoter activity, Fli-1 behaved in a similar fashion to Ets-1 on all constructs examined and activated a mutant CCN2 promoter lacking the Smad response element (data not shown). Collectively, these results suggest that ETS family members activate the CCN2 promoter through a GGAA located within the CCN2 proximal promoter.

### Ets-1, but not Fli-1, potentiates the TGFβ-induction of CCN2

**Figure 2:**
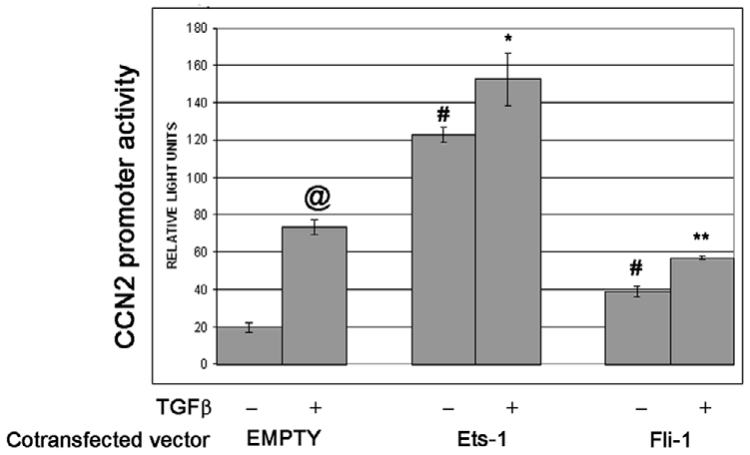
Ets-1, but not Fli-1, enhances the ability of transforming growth factor (TGF)β to induce the CCN2 promoter. A CCN2 promoter/reporter construct driven by nucleotides -805 to +17 of the CCN2 promoter was transfected into fibroblasts in the presence or absence of a 24 h treatment with 4 ng/ml TGFβ1, as indicated. Reporters were co-transfected with empty expression vector, or expression vectors encoding Ets-1 or Fli-1 (0.5 μg expression vector/well) as indicated. Average ± standard deviation (N = 6) is shown. Relative promoter expression is shown. Whereas addition of Ets-1 potentiates the TGFβ1 induction of CCN2 promoter activity (*p < 0.05), Fli-1 limits the TGFβ induction of CCN2 promoter activity relative to the induction of CCN2 promoter activity with TGFβ in the presence of co-transfected empty expression vector (**p < 0.05). Both Fli-1 and Ets-1, however, activate the CCN2 promoter in the absence of TGFβ1 (^#^*p *< 0.05). TGFβ induces the CCN2 promoter in the absence of overexpressed transcription factor (^@^*p *< 0.05) Average ± standard deviation (N = 6) of a representavtive experiment is shown. Reporter activity was adjusted for differences in transfection efficiencies among samples using a control β-galactosidase expression vector.

Previously, we showed that the sequence GGAA was involved with the differential ability of the CCN2 promoter to respond to TGFβ in fibroblasts, but not keratinocytes [9]. In addition, a specific protein enriched in fibroblast nuclear extracts bound nucleotides -126 to -77 of the CCN2 promoter and, hence, was likely to contribute to the fibroblast-specific regulation of CCN2 [9]. However, the identity of this protein was not determined in the prior study. To explore the relative contributions of Ets-1 and Fli-1 to the induction of the CCN2 promoter by TGFβ, NIH 3T3 fibroblasts were co-transfected with the full-length CCN2 promoter/SEAP reporter construct and empty expression vector, or expression vector encoding Ets-1 or Fli-1. Cells were treated with TGFβ1 (4 ng/ml, 24 h), and relative CCN2 promoter activities were assessed (Figure 2). As shown in Figure 1, fibroblasts transfected with Ets-1 or Fli-1 alone showed a significant increase in CCN2 promoter activity. As anticipated, TGFβ activated the CCN2 promoter. However, a further increase in CCN2 promoter activity in response to TGFβ was noted in cells transfected with Ets-1. In contrast, transfection of fibroblasts with an expression vector encoding Fli-1 significantly attenuated the response of the CCN2 promoter to TGFβ (Figure 2). Collectively, these results are consistent with the notion that Ets-1 potentiates the TGFβ activation of the CCN2 promoter, whereas Fli-1 restricts the activation of the CCN2 promoter to TGFβ1 (Figure 2).

### Ets-1 and Smad3 synergize to activate the CCN2 promoter in a PKC-independent fashion

**Figure 3:**
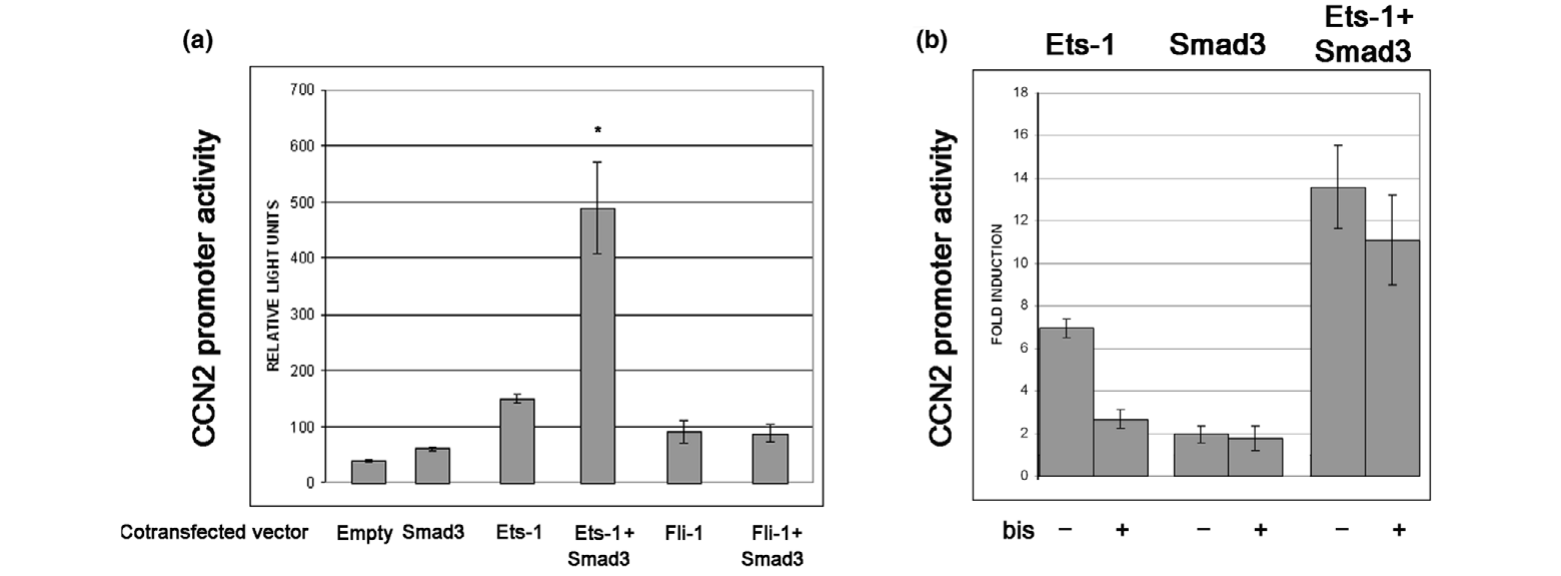
Ets-1 synergizes with Smad3 to activate the CCN2 promoter. **(a) **Ets-1, but not Fli-1, synergizes with Smad3 to activate the CCN2 promoter. A CCN2 promoter/reporter construct driven by nucleotides -805 to +17 of the CCN2 promoter was transfected into fibroblasts in the presence of empty expression vector or expression vector encoding Ets-1, Fli-1 or Smad3, as indicated. After a serum starvation step of 24 h, cells were incubated for an additional 24 h in the presence or absence of 4 ng/ml TGFβ1, as indicated. Co-transfection of Ets-1 and Smad3 (*p < 0.05), but not co-transfection of Fli-1 and Smad3, significantly potentiates activation of the CCN2 promoter in comparison with transfection of either Ets-1 or Fli-1 alone. Average ± standard deviation (N = 6) of a representative experiment is shown. Relative expression is shown. **(b) **Protein kinase C (PKC) is not required for Ets-1/Smad3 synergy. Addition of the general PKC inhibitor bisindolylmaleimide I (bis; 10 μM) blocks the ability of Ets-1 to activate the CCN2 promoter. Conversely, addition of bisindolylmaleimide I has no effect on the ability of Smad3 to activate the CCN2 promoter, or on the synergistic activation of the CCN2 promoter by both Smad 3 and Ets-1. Thus, the presence of excess Smad 3 allows Ets-1 to overcome a requirement for PKC, and permits the activation of the CCN2 promoter in the absence of PKC. Average ± standard deviation (N = 6) is shown. Fold induction by Ets-1, Smad3 or Ets-1/Smad3 is shown, relative to empty control expression vector. Reporter activity was adjusted for differences in transfection efficiencies among samples using a control β-galactosidase expression vector.

Previously, we have shown that Smad3 is required for the TGFβ-induction of CCN2 and that Smad3 activates the CCN2 promoter [19]. To elucidate the effect of Smad3 on the ability of Ets-1 and Fli-1 to regulate the CCN promoter, we next co-transfected the CCN2 promoter/reporter construct with expression vectors encoding Ets-1 or Fli-1 individually, or together with an expression vector for Smad3 (Figure 3a). Co-transfection of either Ets-1, Fli-1 or Smad3 individually modestly activated the CCN2 promoter. However, a marked synergistic activation of the CCN2 promoter was observed in the presence of both Ets-1 and Smad3. Conversely, such synergistic activation was not found upon co-transfection of Smad 3 with Fli-1, suggesting that the different Ets family members show differential use of Smad3 as a co-activator.

The ability of Ets-1 to increase target gene expression may depend on PKC [22], a pathway previously shown to be involved in CCN2 expression [9,10]. Conversely, Smad3-dependent expression of CCN2 is independent of PKC [9,10]. To explore the nature of the synergy between Smad3 and Ets-1 in the activation of the CCN2 promoter, we examined the effect of pharmacological inhibition of PKC, using the general PKC inhibitor bisindolylmaleimide I, to affect the ability of Ets-1, either in the presence or absence of Smad3, to activate the CCN2 promoter (Figure 3b). As anticipated, inhibition of PKC, at a concentration generally used in fibroblasts and shown to be specific for PKC isoforms [9,10,20], significantly reduced the activation of the CCN2 promoter by Ets-1. Intriguingly, the synergistic activation of the CCN2 promoter observed when Smad3 and Ets-1 were overexpressed together was not blocked by bisindolylmaleimide I, suggesting that the presence of Smad3 permits Ets-1 to overcome a requirement for PKC. These results are consistent with the notion that Smads act to potentiate the activity of basal transcription factors [23], and suggest that Smad3 enables Ets-1 to overcome a requirement for PKC in the activation of target promoters.

### Ets-1 and Fli-1 bind the CCN2 promoter

**Figure 4:**
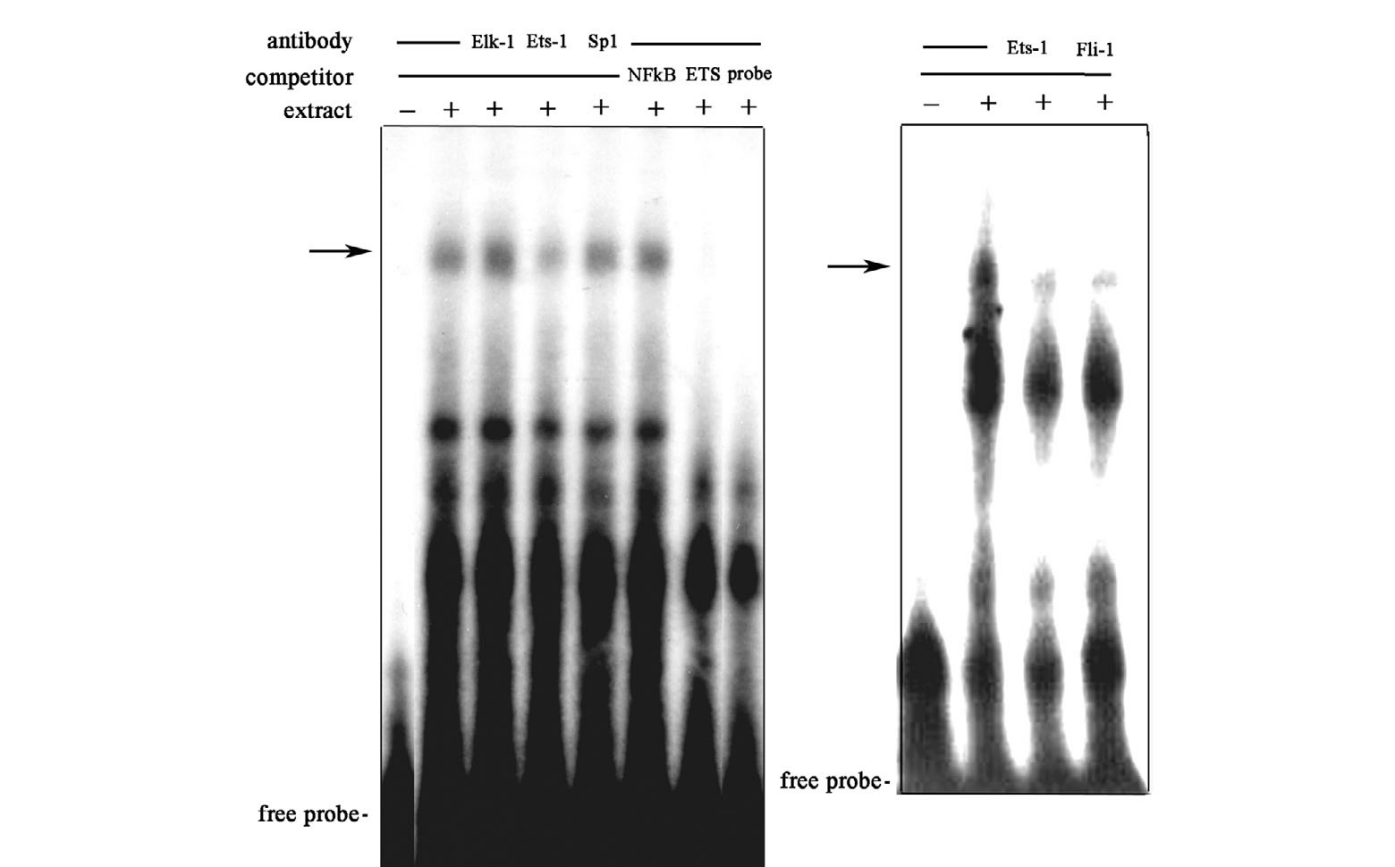
Ets-1 and Fli-1 bind the CCN2 promoter. A double-stranded oligomer corresponding to the -126 to -77 segment of the CCN2 promoter was used in a gel shift assay with NIH 3T3 fibroblast nuclear extract (5 μg) in the presence or absence of 100-fold molar excess of specific competitor corresponding to unlabeled probe (probe), or competitors corresponding to a consensus ETS (ETS) or NFκB binding site, or a 1 h pre-incubation with specific anti-Ets-1 antibody (Ets-1), anti-fli-1 (Fli-1), anti-Elk-1 (Elk-1) or anti-Sp1 (Sp1) antibody. Location of the free probe and shifted Ets-1 containing complex (arrow) are indicated. Representative gel shift assays are shown (N = 3).

To further establish the role of Ets-1 in *CCN2 *gene expression, we determined if endogenous Ets-1 bound the TEF/Ets site of the CCN2 promoter. We performed gel shift assays using nuclear extracts prepared from NIH 3T3 fibroblasts and labeled oligonucleotide containing nucleotides -126 to -77 of the CCN2 promoter. Confirming our previous study where we precisely mapped the nucleotides in this region required for CCN2 promoter activity and protein binding in a gel shift assay [9], a specific DNA/protein complex formed whose presence was abolished by competition with unlabeled probe (Figure 4). A double-stranded oligomer bearing a consensus ETS binding element, but not with a consensus NFκB element, competed for protein binding to the CCN2 promoter (Figure 4). Furthermore, formation of the specific protein-DNA complex was reduced by pre-incubation of binding mixture for 1 hour with a specific anti-Ets-1 and anti-Fli-1 antibody, but not anti-Elk-1 or anti-Sp1 antibodies, prior to addition of probe. Collectively, our results suggest that Ets-1 and Fli-1 bind between nucleotides -126 to -77 of the CCN2 promoter, probably as an oligomer.

### Ets-1 is required for TGFβ-induced CCN2 expression

**Figure 5:**
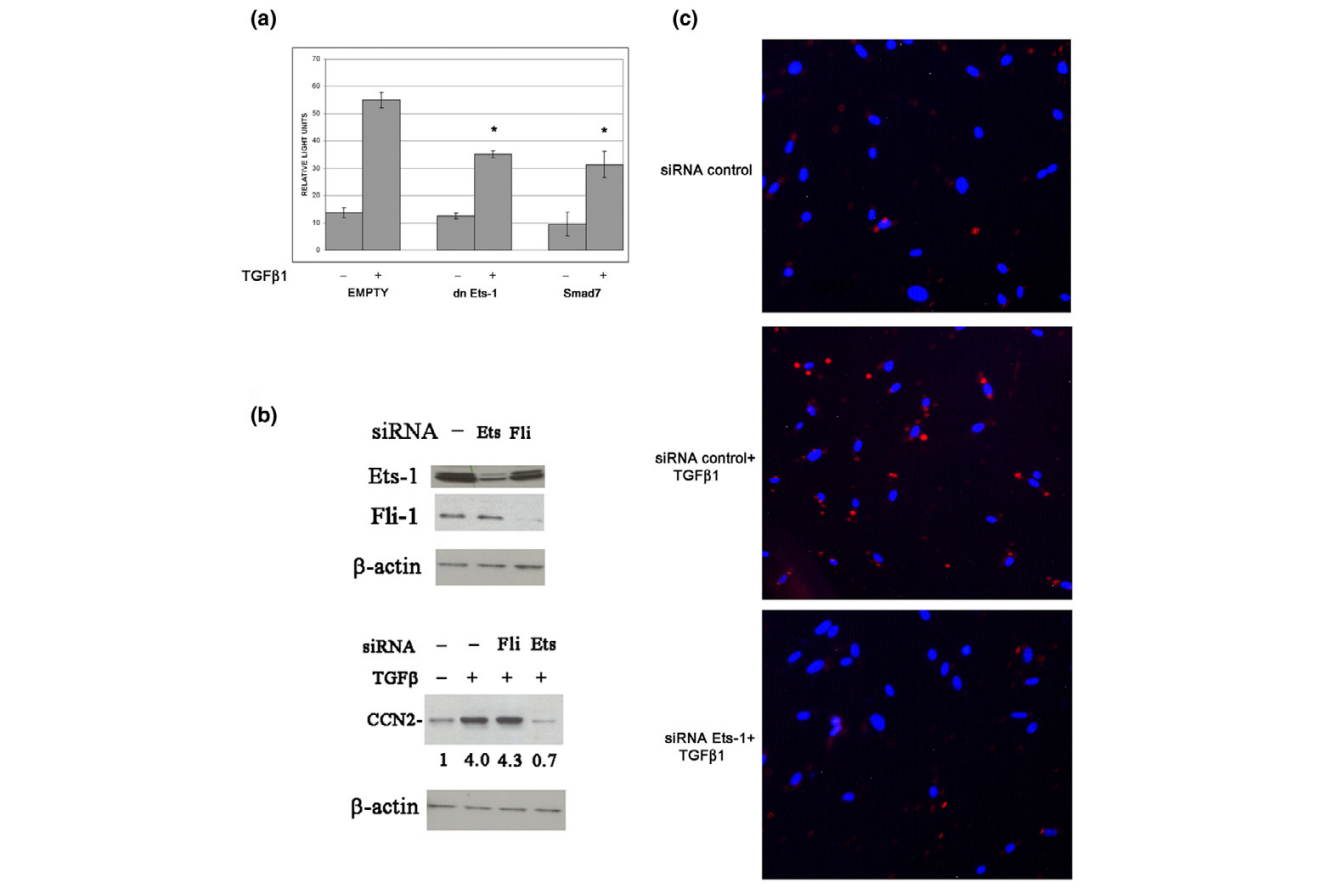
Ets-1 is required for the transforming growth factor (TGF)β induction of CCN2. **(a) **Dominant negative Ets-1 blocks the TGFβ induction of the CCN2 promoter. A CCN2 promoter/reporter construct driven by nucleotides -805 to +17 of the CCN2 promoter was transfected into fibroblasts along with empty expression vector or expression vector encoding dominant negative Ets-1 or Smad7, as indicated. Following serum starvation for 24 hours, cells were incubated in the presence or absence of 4 ng/ml TGFβ1 for 24 h, as indicated. Average ± standard deviation (N = 6) is shown (*p < 0.05). **(b) **Small interfering RNA (siRNA) recognizing Ets-1 mRNA suppresses the TGFβ induction of CCN2. Western blot analysis; fibroblasts were transfected either with control siRNA or siRNA recognizing Ets-1 or Fli-1 mRNAs. Following a serum starvation step of 24 h, cells were incubated in the presence or absence of a 4 ng/ml TGFβ1 for 24 h, as indicated. Proteins were blotted onto nitrocellulose, Membranes were probed with anti-Ets-1, anti-Fli-1 or anti-CCN2 antibodies, as indicated. Values below CCN2 western blot indicate relative amounts of CCN2 protein as determined by densitometry relative to actin. **(c) **siRNA recognizing Ets-1 mRNA suppresses the TGFβ-induction of CCN2. Immunofluorescence analysis; fibroblasts were transfected either with control siRNA or siRNA recognizing Ets-1. After a serum starvation step of 24 h, cells were incubated in the presence or absence of 4 ng/ml TGFβ1 for 24 h, as indicated. Cells were then fixed in paraformaldehyde, and CCN2 was detected with an anti-CCN2 antibody followed by incubation with an appropriate Texas Red-conjugated secondary antibody (red). Cells were costained with DAPI to detect nuclei (blue).

To further investigate the specific contribution of Ets-1 in mediating the TGFβ induction of the CCN2 promoter, we assessed whether overexpression of dominant negative Ets-1 could suppress the response of the CCN2 promoter to TGFβ. We found that, compared to co-transfection of empty expression vector, co-transfection of expression vector encoding dominant negative Ets-1 significantly suppressed the ability of the CCN2 promoter to respond to TGFβ (Figure 5a). Consistent with our previous observations [19], overexpression of Smad7 caused a reduction in CCN2 promoter activation by TGFβ confirming the involvement of the Smad pathway in the TGFβ-induction of CCN2. To further investigate the consequences of eliminating Ets-1 on CCN2 expression, we introduced specific siRNA recognizing Ets-1, Fli-1 or a control siRNA into fibroblasts and exposed cells to TGFβ for 24 hours. Western blot analysis revealed that Ets-1 siRNA and Fli-1 siRNA were effective at reducing Ets-1 or Fli-1 protein expression, respectively (Figure 5b). However, only Ets-1 siRNA was able to reduce CCN2 expression (Figure 5b). As cellular CCN2 is readily detected in the Golgi apparatus of mesenchymal cells [3,10] we assessed CCN2 expression using indirect immunofluorescence analysis with an anti-CCN2 antibody. Cells transfected with Ets-1 siRNA, but not control siRNA, showed reduced CCN2 expression in response to TGFβ (Figure 5c).

Collectively, these data suggest that a functional binding motif for the ETS family of transcription factors resides in the CCN2 promoter, corresponding to one part of the element of the CCN2 promoter necessary and sufficient to respond to TGFβ [9]. However, our results suggest that Ets-1, but not Fli-1, are required for the ability of the CCN2 promoter to respond to TGFβ. To our knowledge, this is a novel, functional divergence within the ETS family, and points to the potential of selective use of Ets family members for particular cellular responses.

## Discussion

CCN2 is induced by TGFβ in adult mesenchymal cells in a Smad-dependent fashion, but is constitutively overexpressed in diseases of excessive matrix production and remodeling, including cancer, fibrosis and arthritis [6]. The expression of CCN2 can be either dependent or independent of exogenous TGFβ [6,19,24,25]. Previously, we showed a sequence in the CCN2 promoter, GAGGAATGG, was required for basal and TGFβ-induced CCN2 expression [9]. In this report, we identify that this element responds to the ETS family of transcription factors, which bind the consensus sequence GGAA [26,27]. The TGFβ response element of the CCN2 promoter has several components, including a Smad element and a GAGGAATGG element, that together are capable of conferring TGFβ-responsiveness to a heterologous promoter [9]. Consistent with the notion that the TGFβ-induction of CCN2 requires Smads, TGFβ does not induce CCN2 protein expression in *Smad3*-/- embryonic fibroblasts [19]. In this report, we show that Ets-1 and Smad3, but not Fli-1 and Smad3, cooperate to activate the CCN2 promoter in the absence of added TGFβ, emphasizing the functional significance of Ets-1 and Smad3 interactions. In addition, we show that Ets-1 is required for the TGFβ induction of CCN2, as dominant negative Ets-1 and siRNA recognizing Ets-1 attenuate the ability of TGFβ to induce the CCN2 promoter activity and protein expression in fibroblasts. Thus, for the first time, our data identify a role for ETS family members, and Ets-1, in the regulation of CCN2 expression.

Smads interact with other transcription factors to form an active transcriptional complex on promoters [23]. That Smad3 and Ets-1 synergize to activate CCN2 expression suggests that Smad3 and Ets-1 functionally interact. Indeed, it has been recently shown that Smad3 and Ets-1 co-immunoprecipitate and act to form a transcriptionally active complex with the transcriptional cofactor p300 [28]. In this latter report, it was shown that Smad3 and Ets-1 also interact with the basal transcription factor Sp1, and that inhibition of Sp1 with mithramycin blocked the TGFβ induction of tenascin-C [28]. Consistent with this notion, we have shown that whereas the Sp1 element of the CCN2 promoter is not necessary for the TGFβ response element to act as an enhancer when placed in front of a heterologous promoter [9,25], the Sp1 inhibitor mithramycin blocks the TGFβ-mediated induction of CCN2 protein in fibroblasts [24]. Our studies using an anti-Sp1 antibody revealed that Sp1 was not present in the protein complex binding to the Ets element of the CCN2 promoter, indicating that chromatin looping is likely to be involved in the interaction between Ets and Sp1. It is interesting to note that within the context of the experiments performed in this present study, transfected Smad3 was able to induce the CCN2 promoter to greater effect than TGFβ ligand, emphasizing that endogenous Smad levels are not likely to be saturating.

The different effects of Ets-1 and Fli-1 on controlling CCN2 promoter activity is intriguing in light of the fact that approximately 25 human ETS proteins have been identified, all of which share a highly conserved DNA binding domain that interacts with the core DNA target GGAA/T [12,13]. It has been hypothesized that the existence of many different ETS factors suggests that individual Ets members may have unique roles [12,13]. Subtle differences in target sites or their own expression in tissues, and differential response to external signals may contribute to distinct functions, activating or repressing target gene expression – either basally or in response to growth factors – depending on a constellation of ETS factors that compete for binding to ETS binding elements [28-38]. Some recent data have shown that ETS family members contribute to the regulation of genes that mediate matrix remodeling, cell migration and cancer progression, including those controlling cell proliferation, adhesion cell survival, invasion, and signaling [31-38]. Several recent studies have focused in particular on the potentially divergent roles of Fli-1 and Ets-1 in providing a balance between tissue homeostasis and repair/remodeling [22,30,34-37]. Consistent with this notion, both Ets-1 and Fli-1 activate the promoters of matrix metalloproteinases [22,34-37], enzymes involved with degrading matrix and promoting cell migration. Similarly, Ets-1 activates tenascin C, an extracellular matrix glycoprotein that promotes cell migration and angiogenesis [32,33], and CCN2, encoded by an immediate-early gene that also promotes cell adhesion and migration and angiogenesis [2,40,41]. Conversely, type I collagen is induced by Ets-1 but repressed by Fli-1 [30,34,42]. In the current study, the induction of the CCN2 promoter in response to TGFβ is reduced by Fli-1, and diminished by dominant negative Ets-1, supporting a divergence in the roles of Ets-1 and Fli-1 in gene regulation. As we observed for CCN2, TGFβ induction of tenascin-C is potentiated by Ets-1; however, the TGFβ-induction of type I collagen is impaired by Ets-1 [30,34,42]. Given that Ets-1 is induced during the early phases of tissue repair [14,38,39] and is overexpressed in tumor stroma, [12,13,41], these results, although albeit using principally promoter-based approaches, collectively suggest that Ets-1 could bias the fibroblast population towards a 'pro-migratory' program in that TGFβ and Ets-1 interactions may bias Ets-1 and TGFβ-responsive genes toward a migratory/adhesive/invasive phenotype. Conversely, at later stages of repair when Ets-1 levels decrease, the effects of TGFβ may switch towards matrix rebuilding, with increased type I collagen resulting in wound closure.

## Conclusion

Our investigation into the mechanism underlying the control of CCN2 regulation in fibroblasts has revealed a role for an ETS binding element within the CCN2 promoter. In particular, we show that the transcription factor Ets-1 contributes to the TGFβ induction of the CCN2 promoter and protein. Ets-1, but not the related Fli-1, synergize with Smad3 in activating the CCN2 promoter, suggesting that the CCN2 promoter can be differentially regulated by different members of the ETS family. Our results point to the complexity underlying CCN2 expression, and are consistent with the notion that different ETS family members can have distinct influences on gene expression in fibroblasts. As CCN2 plays roles in connective tissue pathologies, targeting Ets-1 may be beneficial in alleviating pathologies of tissue remodeling and repair, including cancer, arthritis and fibrosis.

## Abbreviations

DMEM = Dulbecco's modified Eagle's medium; PKC = protein kinase C; SEAP = secreted enhanced alkaline phosphatase; siRNA = small interfering RNA; TGF = transforming growth factor; TEF = transcription enhancing factor.

## Competing interests

The authors declare that they have no competing interests.

## Authors' contributions

JvB and LK performed cell culture, transfection, promoter analysis, immunofluorescence and siRNA studies. JR performed the gel shift assay. SB helped write the manuscript. AL performed the gel shift assay, prepared the manuscript and designed the experiments.
